# Supplementary material for: Taming nitroformate through encapsulation with nitrogen-rich hydrogen-bonded organic frameworks
Source: Nat Commun. 2021 Apr 9;12:2146. doi: 10.1038/s41467-021-22475-8 (PMC8035193; doi:10.1038/s41467-021-22475-8)
Supplement: Supplementary file 1 — Supplementary Information [file 41467_2021_22475_MOESM1_ESM.pdf]

Supplementary Information

Taming Nitroformate through Encapsulation with Nitrogen-Rich Hydrogen-Bonded Organic Frameworks

Jichuan Zhang,[1,2,3] Yongan Feng,[4] Richard J. Staples,[5] Jiaheng Zhang,[2,3]* Jean'ne M. Shreeve[1]*

[1]Department of Chemistry, University of Idaho, Moscow, Idaho 83844-2343, USA.
[2]Research Centre of Flexible Printed Electronic Technology, Harbin Institute of Technology, Shenzhen, 518055, China.
[3]Zhuhai Institute of Advanced Technology Chinese Academy of Sciences, Biomaterials Research Center, Zhuhai, 519003, China.
[4]School of Environmental and Safety Engineering, North University of China, Taiyuan 030051, China
[5]Department of Chemistry, Michigan State University, East Lansing, MI 48824, USA.

Corresponding Authors: Jean'ne M. Shreeve, Jiaheng Zhang
Email: jshreeve@uidaho.edu, jiahengzhang@hit.edu.cn.

	Page
Supplementary Single crystal information	S3
Supplementary Statistic works of NF-based compounds	S4
Supplementary Thermogravimetric analysis (TG)	S8
Supplementary Computation and properties	S8
Supplementary References	S13

Supplementary Single crystal information

Crystal Structure Analysis.

Cocrystals **HOF-NF.** Single red block crystals of JS620E were used as received. A suitable crystal with dimensions 0.27 × 0.15 × 0.07 mm^3 was selected and mounted on nylon loops with Paratone oil. Data were collected using a XtaLAB Synergy, Dualflex, HyPix diffractometer equipped with an Oxford Cryosystems low-temperature device, operating at T = 100.01(10) K.

Supplementary Table 1. Crystallographic data and structure refinement parameters of **HOF-NF.**

**Crystal**	**HOF-NF**
CCDC	2027045
Empirical formula	$C_{10}H_{19}N_{23}O_6$
Formula mass	577.48
Crystal system	triclinic
Space group	P-1
Z	4
a (Å)	6.8812
b (Å)	15.2928
c (Å)	20.4463
α (°)	95.394
β (°)	90.462
γ (°)	93.452
Volume ($Å^3$)	2138.02
D_{calc} (g cm^{-3})	1.732
Temperature (K)	100
F(000)	1152.0
h, k, l	8,19,25
μ (cm-1)	1.256
R_1 [I > 2σ(I)]	0.0524
Completeness to theta full (%)	0.955
wR_2 (all data)	0.1581
S on *F2*	1.038

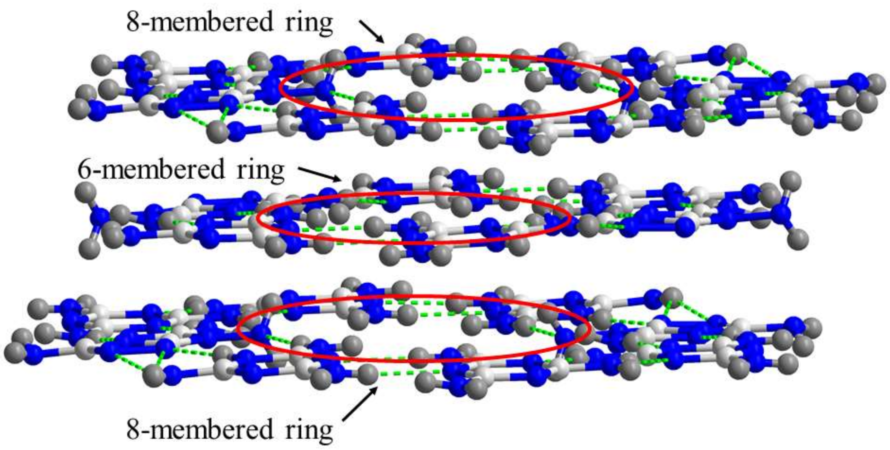

Supplementary Fig 1. Arranged rings of adjacent layers

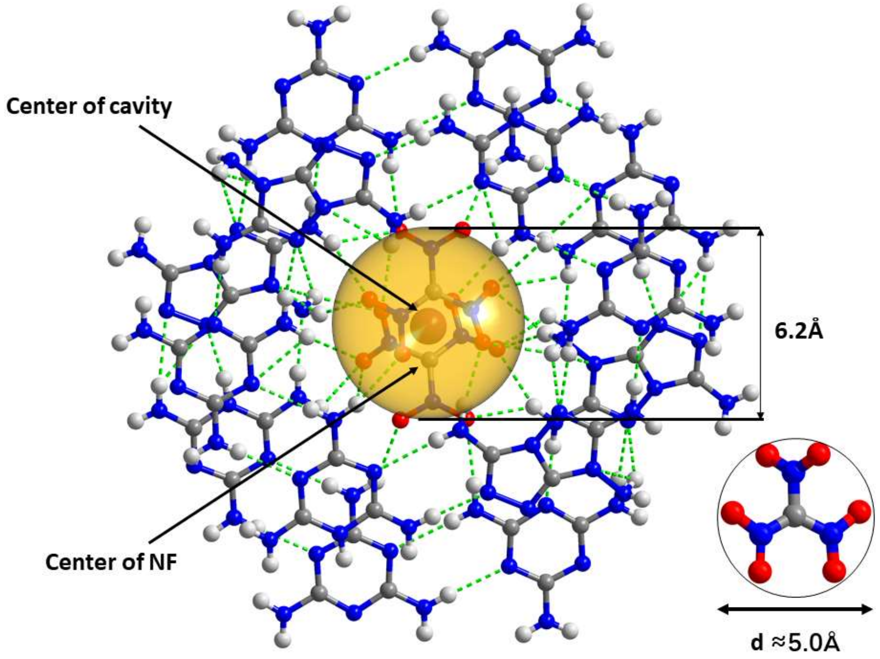

Supplementary Fig 2. Locations of NF anions in HOF-NF

Supplementary Statistic works of NF-based compounds

Almost all compounds (1-34) based on NF were found in the literature.[2-13] They are listed in Supplementary Fig 1, and their decomposition temperatures are listed in Supplementary Table 2. From Supplementary Table 2, it is obvious that except for compounds 15, 21 and 28, the decomposition temperatures of all compounds based on NF are lower than 150 °C.

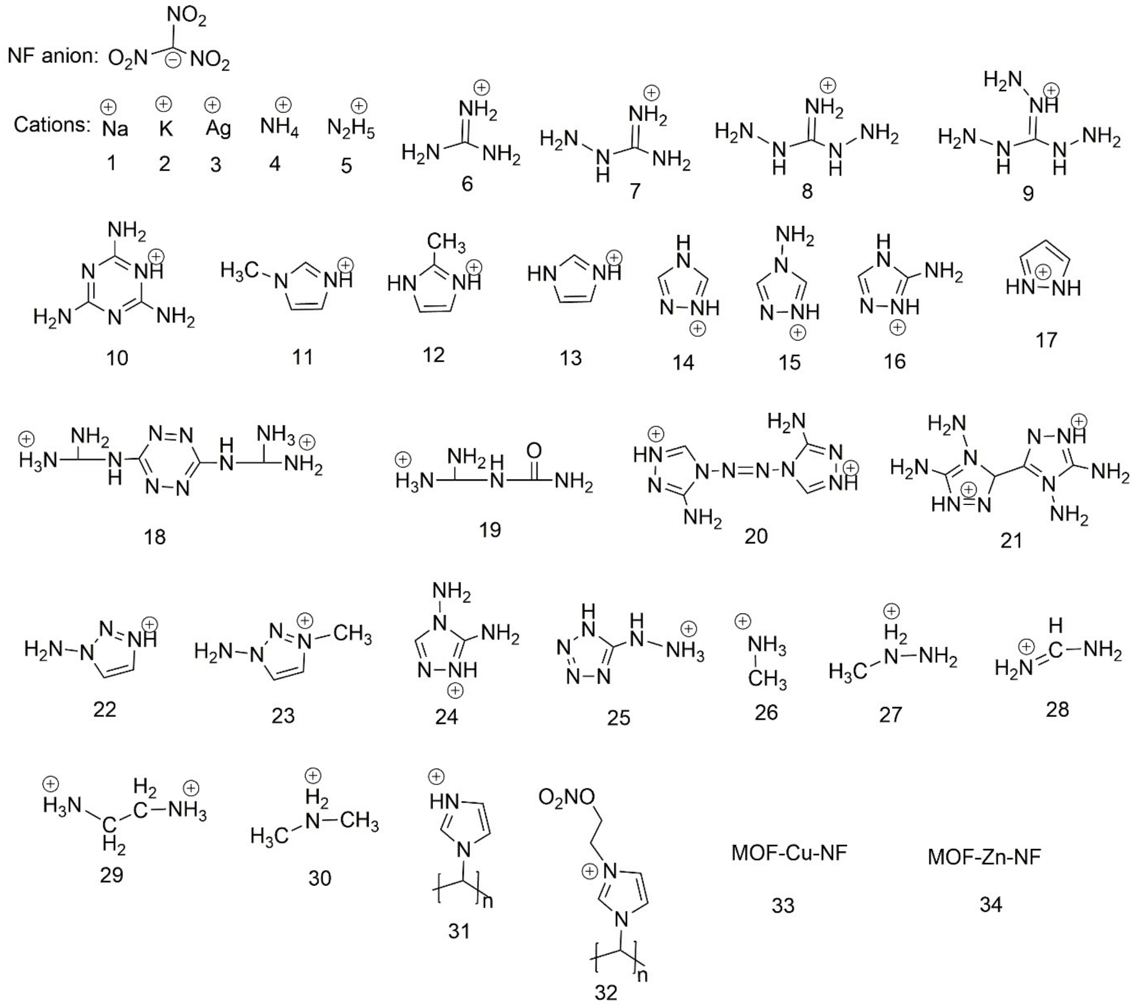

Supplementary Fig 3. Structural formulas of reported NF-based compounds

Supplementary Table 2. Decomposition temperatures of compounds based on nitroformate

Compound	Decomposition (°C)	Note	Compound	Decomposition (°C)	Note
**1**	140	Ref 11	**21**	94	Ref 13
**2**	75	Ref 12	**22**	~130	Ref 7 #b
**3**	65	Ref 12	**23**	~150	Ref 7 #c
**4**	116	Ref 2	**24**	103	Ref 5
**5**	123	Ref 2	**25**	~120	Ref 6 #d
**6**	113	Ref 2	**26**	95	Ref 4
**7**	71	Ref 2	**27**	115	Ref 4
**8**	82	Ref 2	**28**	166	Ref 4 #e
**9**	105	Ref 2	**29**	105	Ref 4
**10**	143	Ref 2	**30**	110	Ref 4
**11**	83	Ref 3	**31**	106	Ref 10
**12**	98	Ref 3	**32**	83	Ref 10
**13**	104	Ref 3	**33**	141	Ref 9

**14**	90	Ref 3	**34**	150	Ref 9
**15**	167	Ref 3 [#a]			
**16**	74	Ref 3			
**17**	86	Ref 3			
**18**	100	Ref 3			
**19**	153	Ref 3			
**20**	80	Ref 8			

Note:

[#a]: The decomposition temperature of 15 is 167°C, because in fact, this compound is a cocrystal, which consists of one 4-amino-triazole neutral compound and one 4-amino-triazolate nitroformate; the neutral compound provides many more H-bonds than in other compounds. However, at least one O atom of NF in compound 15 is not surrounded by H-bonds.

[#b] and [#c]: The authors provided the decomposition peaks for all compounds, and they didn't provide the DSC curves of compounds 22 and 23. Based on the DSC curves of other compounds they provided, the decomposition temperatures (onset) of 22 and 23 are estimated to be around 130 and 150 ℃.

[#d]: The authors provided decomposition peaks of all compounds as their decomposition temperatures, but they did not provide the DSC curve of compound 25. According to the compound based on dinitramide, whose decomposition onset is 137 ℃, and its peak is at 188 °C. We believe the decomposition temperature (onset) for compound 25 should be lower than the dinitramide analogue, and its decomposition onset is estimated to be around 120℃.

[#e]: Although the authors provided the DSC curve of compound 28 in the cited reference, and the decomposition temperature (onset) should be about 167°C, not 171°C. In addition, its DSC curve is not flat, and from about 110°C, it begins to decompose slightly. The crystal structure of 28 shows that two O atoms of nitroformate in compound 28 aren't bonded by H atoms, and the slight decomposition of 28 may be due to the two unbonded O atoms of nitroformate.

Among these compounds, except for compound 4, the severely disordered one, the crystal structures of compounds 5-10, 15, 18, 21, 24, 26-30, 33-34 were found, and the number of H bonds surrounding NF anions are drawn in Supplementary Fig 4 in the order of H bonds from more to less. The setting range for searching H-bonds is 90°< angle <180°, and the setting length for searching H-bonds is 1.5Å< angle <2.6Å. From Supplementary Fig 4, we can see that 1) except for NF1 and NF2, whose O were all fixed by surrounding H-bonds, all remaining compounds have at least one O atom, which is not fixed by H-bonds; 2) compounds 33 and 34 are special, because there are no H-bonds around them, and their decomposition temperatures are 141 and 150°C, respectively. Notably, the strengthened MOFs' frameworks stabilize them very well, which also explains that the HOF framework could stabilize NF anions, even though the strength of an HOF's framework is smaller than those in MOFs.

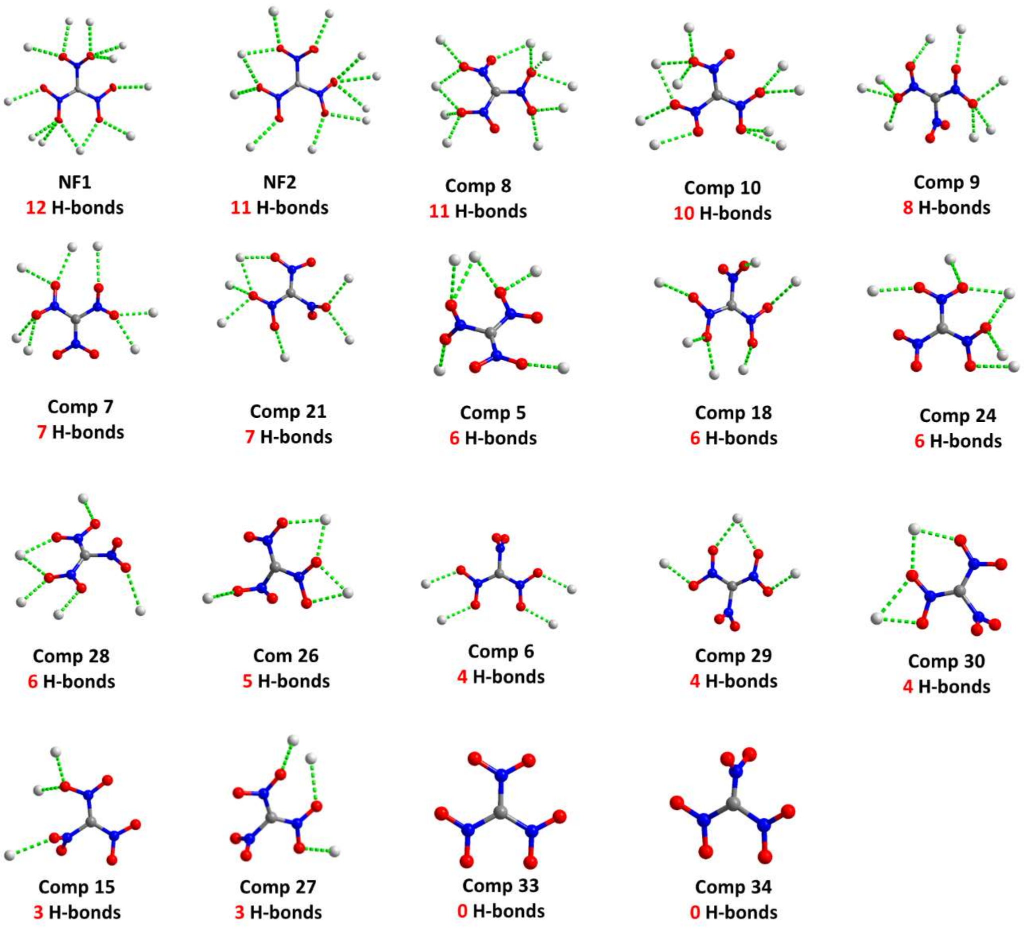

Supplementary Fig 4. Number of H-bonds surrounding NF among reported NF-based compounds.

Supplementary Thermogravimetric analysis (TGA)

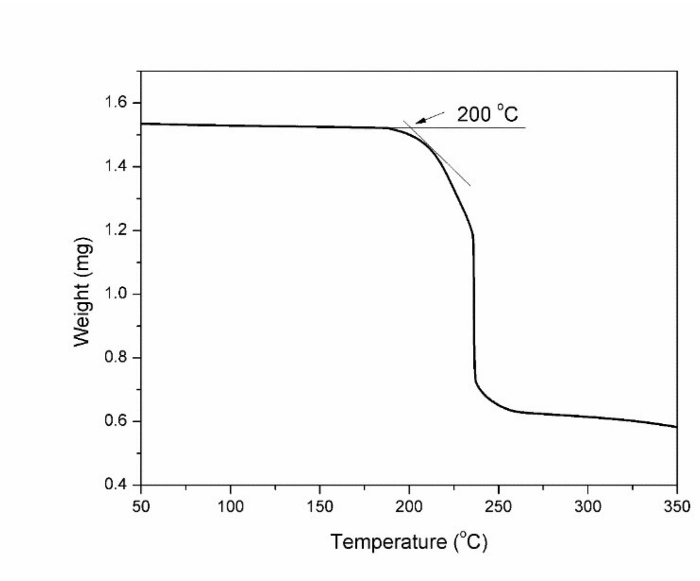

Supplementary Fig 5. The thermogravimetric analysis (TG) thermal behavior of HOF-NF (5 °C min^{-1} in N_2 atmosphere)

The thermogravimetric analysis (TG) thermal behavior of HOF-NF was recorded by a TAQ50, and the decomposition temperature (200 °C) was obtained according to literature.[14]

Supplementary Computation and properties

Noncovalent interaction (NCI): According to the literature,[15,16] Noncovalent interaction (NCI) surrounding NF1 and NF2 were calculated. The gjf files of NF1 and NF2 were derived from the crystal file of HOF-NF using Mercury software. Because of that the positions of the H atoms were not determined when solving the single crystal structure. Hence, the positions of the H atom need to be optimized. Additionally, all nonhydrogen atoms need to be restrained. Here, the PM7 method was employed - see the original input files of NF1 and NF2 (gjf format, because the resulting files of this step are too big, they are not provided.). In order to obtain the functional files for weak interactions in crystal, another DFT calculation (B3LYP/6-311G (d))[17] are needed to calculate the optimized files after the PM7 calculation - see the input files of NF1SP and NF2SP (gjf format) and resulting files (corresponding out files). Finally, the Noncovalent interaction (NCI) plots of NF1 and NF2 were drawn using two softwares: Multiwfn and VMD.[18,19]

Hirshfeld surfaces: The involved software for Hirshfeld surface [16](two-dimensional fingerprint plots) is CrystalExplorer.[20] Among those single crystal structures found according to the literature, compounds 26 and 29 were not suitable for calculating the fingerprint plots

because of the disordered atoms in the crystal structure. From Supplementary Fig 6, we can see that except for compound 15, all the bond strengths (distances) of N (O)···H interactions among these compounds are smaller than those in HOF-NF. Although in compound 15, the bond strength of N···H is as strong as in HOF-NF, the percentage of N···H interactions is 21.3% which is lower than that in HOF-NF (28.0%).

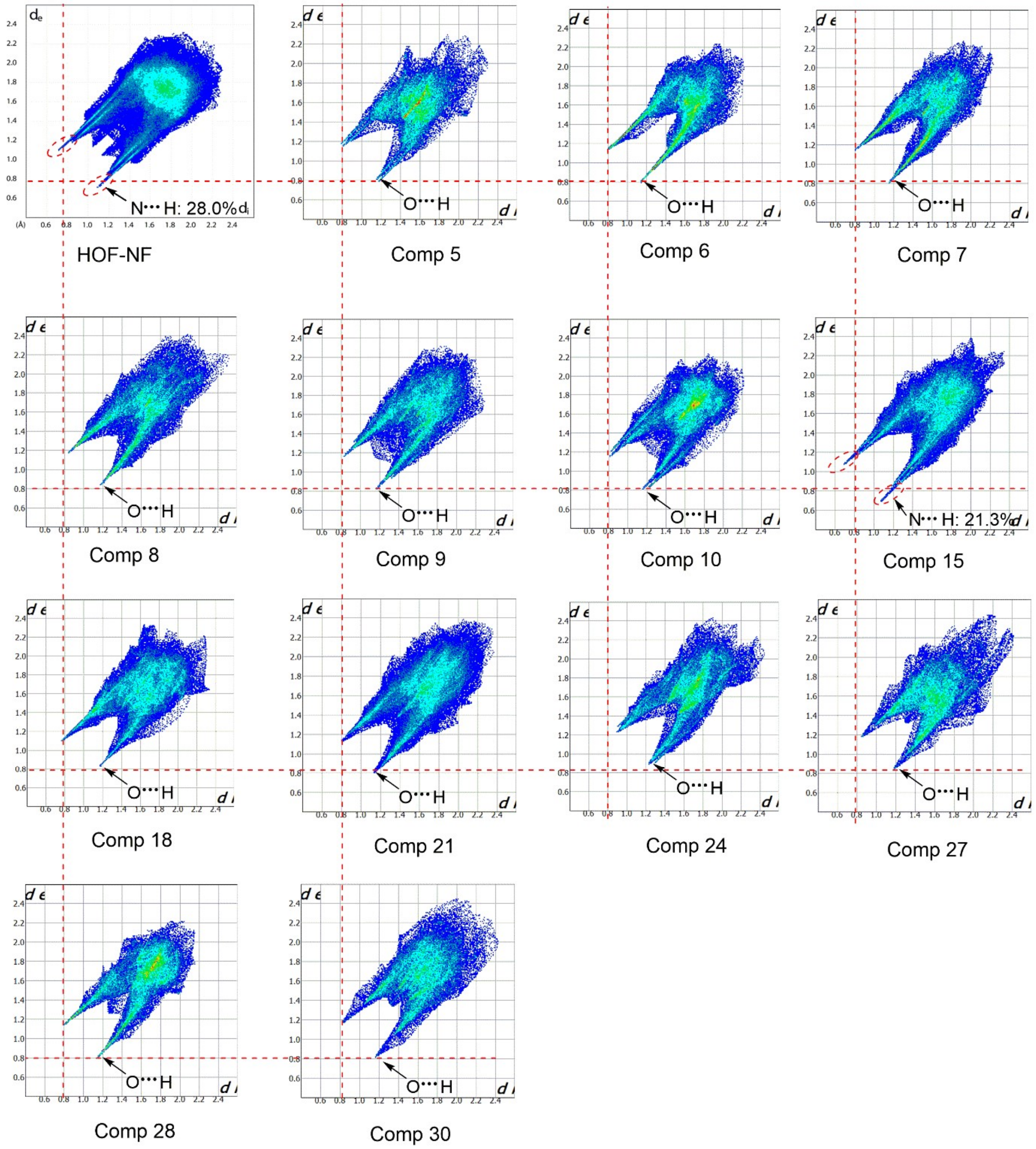

Supplementary Fig 6. Two-dimensional (2D) fingerprint plots of reported NF-based compounds

ESP calculations. The geometries of the unit cells were optimized at the B3LYP/6-31+G**[16,17] level using Gaussian 03 (Revision D.01) suite of scripts.[21] Since the resulting files are too large,

only the 5 original input files (4ATNF, DGNF, FNF, HOF-NF, and MaNF) are provided. The following ESP figures (Supplementary Fig 7-11) of these 5 crystals were drawn using software of Multiwfn and VMD.

Note: blue numbers: minimum values; red numbers: maximum values

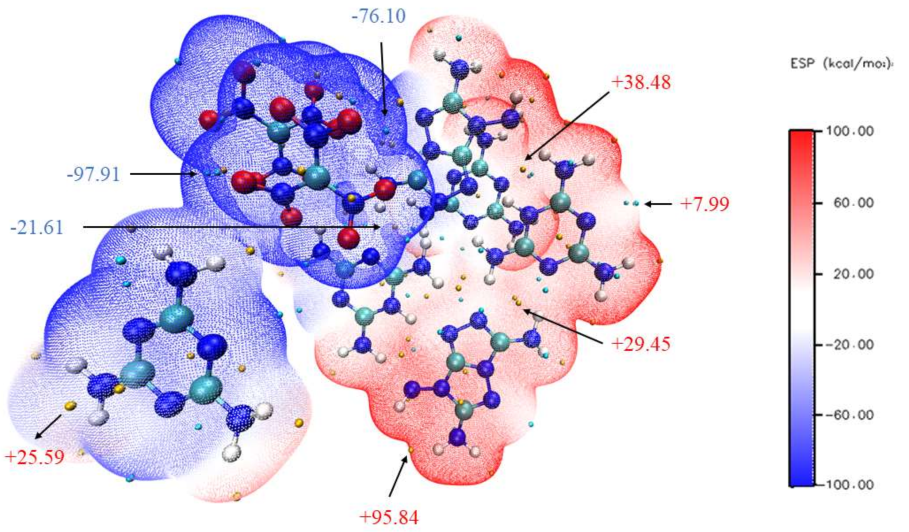

Supplementary Fig 7. ESP of HOF-NF

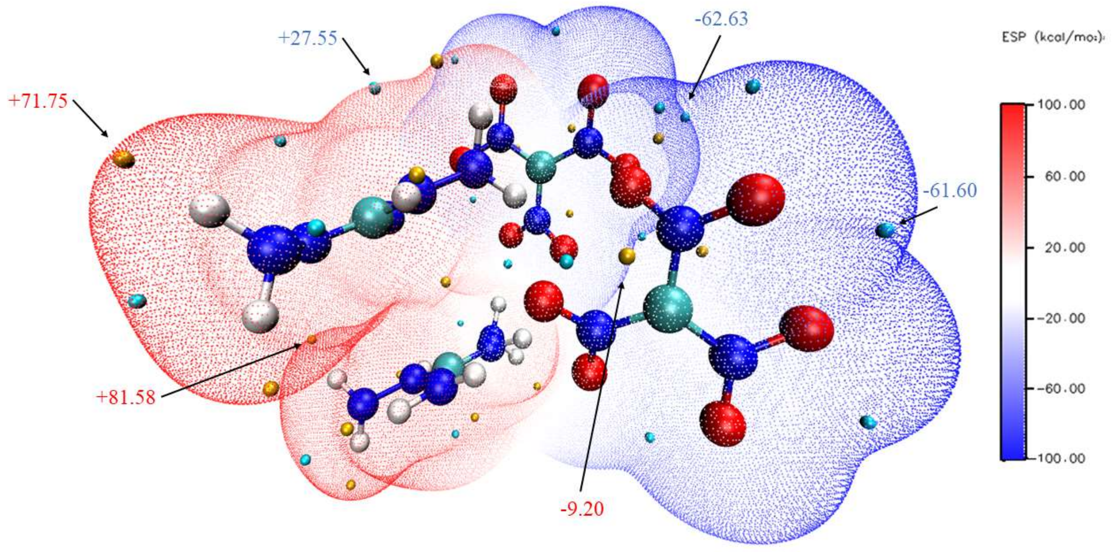

Supplementary Fig 8. ESP of DGNF (diaminoguanidinium)

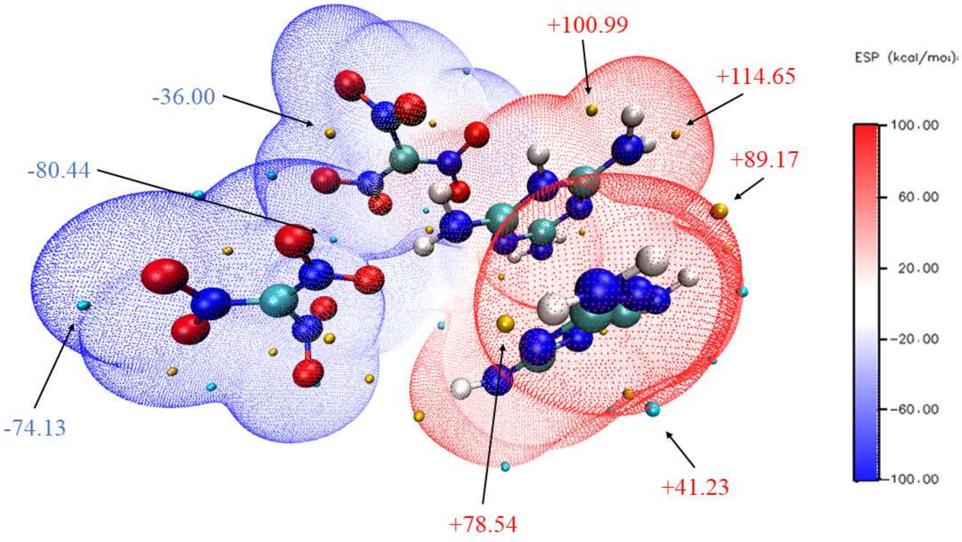

Supplementary Fig 9. ESP of MaNF (melamine nitroformate)

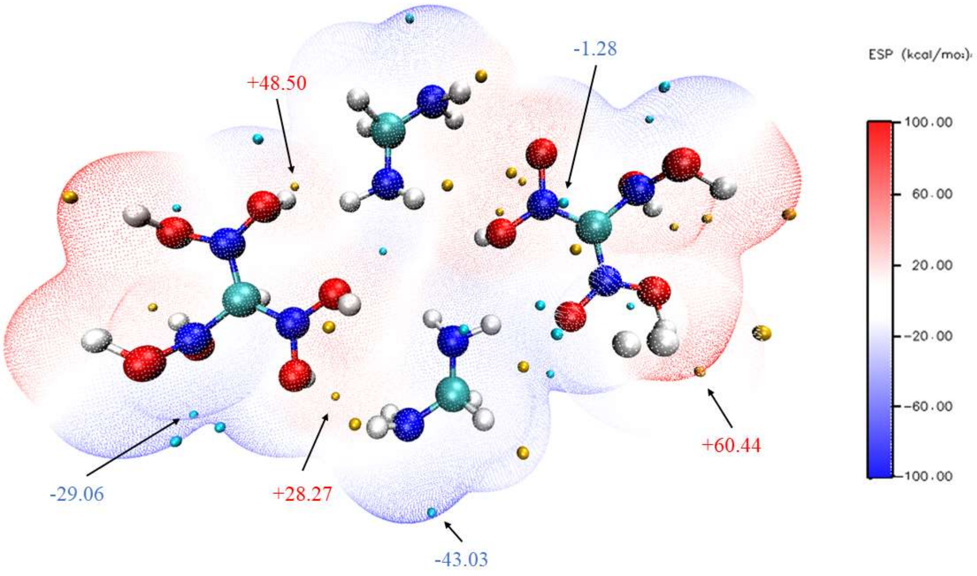

Supplementary Fig 10. ESP of FNF (formamidinium nitroformate)

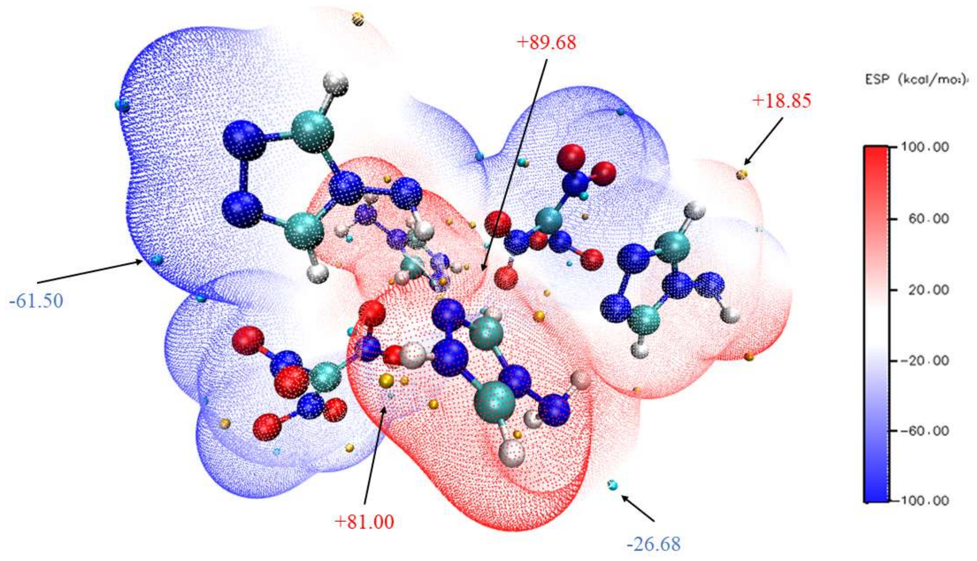

Supplementary Fig 11. ESP of TNF (4-amino-1,2,4-triazolatenitroformate)

Supplementary Table 3. The calculated average ESP of maximum and minimum values

Selected compounds	Average of maximum values kcal mol^{-1}	Average of minimum values kcal mol^{-1}
HOF-NF	39.47	-65.20
DGNF	54.14	-32.23
MaNF	84.92	-63.52
FNF	45.74	-24.46
4ATNF	63.18	-44.09

Heat of formation.

HOF-NF was treated as a cocrystal of melamine nitroformate, melamine and TATOT. Theoretical calculations of the melamine cation were performed by using the Gaussian 03 (Revision D.01) suite of scripts.[21] The geometric optimization and frequency analyses of melamine cation were completed by using the B3LYP functional with the 6-31G(d) basis set. Single energy points were calculated at the MP2/6-31G (d) level of theory (see the original input file in gjf format and the resulting file of melamine cation in com.out format). We have checked that the structure relaxed to a minimum structure of the melamine cation by showing that its frequency is real. According to the literature, the heat of formation of the NF anion, Ma and TATOT in the solid phase are -228.8, -66.1 and 470.5 kJ mol^{-1} (Supplementary Table 4), respectively. The solid-state heat of formation (HOF-NF, ΔfH°) was calculated based on a Born–Haber energy cycle[22] with the following simplified equation for calculation:

ΔfH° (HOF-NF, 298K) = ΔfH° (precursor 1, 298K) + ΔfH° (precursor 2, 298K) + ΔfH° (precursor 3, 298K) – ΔHsub

The heat of sublimation can be estimated using the DFT method with the GGA-RPBE (revised Perdew Burke-Ernzerhof) exchange-correlation functional in Dmol3 program with the basis set of DND.[23-24] The heat of formation of HOF-NF was calculated to be 376.4 kJ mol^{-1}.

Supplementary Table 4. The related heat of formations for several species

Species	Heat of formation (kJ mol^{-1}, 298K)
Ma cation	650.8
NF anion[4]	-228.8
Ma[25]	-66.1
TATOT[1]	470.5

Thermodynamic calculations. The calculated activation energy based on Kissinger and Ozawa methods,[26] and their values of activation energy are 293.4 and 287.2, respectively. The correlation coefficients (*r*2) of these two methods are 0.975 and 0.976, respectively, very close to 1, which indicates that the results are reliable.

Supplementary Table 5. Physicochemical properties of different energetic compounds.

**Comp.**	ρ ^{a} (g cm^{-3})	**T$_d$** ^{b} °C	**E$_a$** ^{c}(kJ mol-1)	**IS**^{d} (J)	**FS** ^{e}(N)	$\Delta_f H^{o\,f}$ (kJ mol^{-1})	**D$_v$** ^{g} (m s^{-1})	**P** ^{h} (Gpa)
**HOF-NF**	1.68	200	293.4/287.2	>40	>360	367.4	7817	21.63
**TNT** [27]	1.65	295	113.8/117.1	15	353	−31.7	6881	19.50
**RDX**[26]	1.80	204	148.5/149.3	7.5	120	85.0	8750	34.1
**TATB**[28]	1.93	350	211.4/211.3	>40	>360	11.0	8179	32.6

^{a} Experimental density at room temperature.^{b} Decomposition temperature (onset). ^{c} Activation energy obtained using Kissinger's / Ozawa's method. Decomposition temperature (onset). ^{d} Impact sensitivity (J). ^{e} Friction sensitivity (N). ^{f} Enthalpy of formation (kJ mol^{-1}). ^{g} Detonation velocity (m s^{-1}). ^{h} Detonation pressure (Gpa).

Supplementary References

1. Yin, P., Zhang, J., Parrish, D. A. & Shreeve, J. M. Energetic fused triazoles–a promising C–N fused heterocyclic cation. *J. Mater. Chem. A* **3**, 8606-8612 (2015).
2. Göbel, M. & Klapötke, T. M. Potassium-, Ammonium-, Hydrazinium-, Guanidinium-, Aminoguanidinium-, Diaminoguanidinium-, Triaminoguanidinium-and Melaminiumnitroformate–Synthesis, Characterization and Energetic Properties. *Z. Anorg. Allg. Chem.* **633**, 1006-1017 (2007).
3. Huang, Y., Gao, H., Twamley, B. & Shreeve, J. M. Synthesis and characterization of new energetic nitroformate salts. *Eur. J. Inorg. Chem.* **2007**, 2025-2030 (2007).
4. Baxter, A. F., Martin, I., Christe, K. O. & Haiges, R. Formamidinium nitroformate: An insensitive RDX alternative. *J. Am. Chem. Soc.* **140**, 15089-15098 (2018).

5. Wu, J.-T., Zhang, J.-G., Yin, X., Cheng, Z.-Y. & Xu, C.-X. 3, 4-Diamino-1, 2, 4-triazole based energetic salts: synthesis, characterization, and energetic properties. *New J. Chem.* **39**, 5265-5271 (2015).
6. Lin, Q.-H., Li, Y.-C., Qi, C., Liu, W., Wang, Y. & Pang, S.-P. Nitrogen-rich salts based on 5-hydrazino-1H-tetrazole: a new family of high-density energetic materials. *J. Mater. Chem. A* **1**, 6776, doi:10.1039/c3ta10503b (2013).
7. Lin, Q.-H., Li, Y.-C., Li, Y.-Y., Wang, Z., Liu, W., Qi, C. & Pang, S.-P. Energetic salts based on 1-amino-1, 2, 3-triazole and 3-methyl-1-amino-1, 2, 3-triazole. *J. Mater. Chem.* **22**, 666-674 (2012).
8. Liu, W., Li, S.-h., Li, Y.-c., Yang, Y.-z., Yu, Y. & Pang, S.-P. Nitrogen-rich salts based on polyamino substituted N,N′-azo-1,2,4-triazole: a new family of high-performance energetic materials. *J. Mater. Chem. A* **2**, 15978-15986, doi:10.1039/c4ta03016h (2014).
9. Du, Y., Su, H., Fei, T., Hu, B., Zhang, J., Li, S., Pang, S. & Nie, F. Structure–Property Relationship in Energetic Cationic Metal–Organic Frameworks: New Insight for Design of Advanced Energetic Materials. *Crystal Growth & Design* **18**, 5896-5903, doi:10.1021/acs.cgd.8b00640 (2018).
10. Wang, B., Feng, Y., Qi, X., Deng, M., Tian, J. & Zhang, Q. Designing explosive poly (ionic liquid) s as novel energetic polymers. *Chem. Eur. J.* **24**, 15897-15902 (2018).
11. Huang, H., Zhang, J., Zhang, T. & Zhang, S. Thermal stability improved by π-π stacking interactions: Synthesis, crystal structure and thermal decomposition of sodium nitroformate. *Journal of Wuhan University of Technology-Mater. Sci. Ed.* **29**, 488-491 (2014).
12. Göbel, M., Klapötke, T. M. & Mayer, P. Crystal structures of the potassium and silver salts of nitroform. *Z. Anorg. Allg. Chem.* **632**, 1043-1050 (2006).
13. Klapötke, T. M., Schmid, P. C., Schnell, S. & Stierstorfer, J. Thermal stabilization of energetic materials by the aromatic nitrogen-rich 4, 4′, 5, 5′-tetraamino-3, 3′-bi-1, 2, 4-triazolium cation. *J. Mater. Chem. A* **3**, 2658-2668 (2015).
14. Wang, Y., Liu, Y., Song, S., Yang, Z., Qi, X., Wang, K., Liu, Y., Zhang, Q. & Tian, Y. Accelerating the discovery of insensitive high-energy-density materials by a materials genome approach. *Nat Commun* **9**, 2444, doi:10.1038/s41467-018-04897-z (2018).
15. Johnson, E. R. *et al.* Revealing noncovalent interactions. *J. Am. Chem. Soc.* **132**, 6498-6506 (2010).
16. Zhang, J., Zhang, Q., Vo, T. T., Parrish, D. A. & Shreeve, J. M. Energetic salts with π-stacking and hydrogen-bonding interactions lead the way to future energetic materials. *J. Am. Chem. Soc.* **137**, 1697-1704 (2015).
17. Grimme, S., Ehrlich, S. & Goerigk, L. Effect of the damping function in dispersion corrected density functional theory. *J. Comput. Chem.* **32**, 1456-1465 (2011)
18. Lu, T., Multiwfn is always in active development, the original paper is J. Comput. Chem., 33, 580-592 (2012).
19. Humphrey W, Dalke A, Schulten K. VMD: visual molecular dynamics. Journal of molecular graphics, 14(1): 33-38 (1996).
20. Wolff, S. K.; Grimwood, D. J.; McKinnon, J. J.; Turner, M. J.; Jayatilaka, D.; Spackman, M. A. CrystalExplorer, version 3.1; University of Western Australia: Crawley, Australia, 2012.
21. Frisch, M. Gaussian 03 Rev. E. 01. *http://www. gaussian. com/* (2004).

22. Medvedev, V. A., Cox, J. & Wagman, D. D. *CODATA key values for thermodynamics*. (Hemisphere Publishing Corporation New York, 1989).
23. Delley, B. An all-electron numerical method for solving the local density functional for polyatomic molecules. *The Journal of chemical physics* **92**, 508-517 (1990).
24. Delley, B. From molecules to solids with the DMol 3 approach. *The Journal of chemical physics* **113**, 7756-7764 (2000).
25. https://webbook.nist.gov/chemistry/form-ser/
26. Lee, J.-S., Hsu, C.-K. & Chang, C.-L. A study on the thermal decomposition behaviors of PETN, RDX, HNS and HMX. *Thermochim. Acta* **392**, 173-176 (2002).
27. Li, J. S., Chen, J. J., Hwang, C. C., Lu, K. T. & Yeh, T. F. Study on Thermal Characteristics of TNT Based Melt-cast Explosives. *Propellants. Explos. Pyrotech* **44**, 1270-1281 (2019).
28. Tang, Y., He, C., Imler, G. H., Parrish, D. A. & Shreeve, J. n. M. Aminonitro Groups Surrounding a Fused Pyrazolotriazine Ring: A Superior Thermally Stable and Insensitive Energetic Material. *ACS Appl. Energy Mater* **2**, 2263-2267 (2019).
